# Comparative SNP and Haplotype Analysis Reveals a Higher Genetic Diversity and Rapider LD Decay in Tropical than Temperate Germplasm in Maize

**DOI:** 10.1371/journal.pone.0024861

**Published:** 2011-09-15

**Authors:** Yanli Lu, Trushar Shah, Zhuanfang Hao, Suketoshi Taba, Shihuang Zhang, Shibin Gao, Jian Liu, Moju Cao, Jing Wang, A. Bhanu Prakash, Tingzhao Rong, Yunbi Xu

**Affiliations:** 1 Maize Research Institute, Sichuan Agricultural University, Wenjiang, Sichuan, China; 2 International Crops Research Institute for the Semi-Arid Tropics, Patancheru, Hyderabad, Andhra Pradesh, India; 3 Institute of Crop Sciences, Chinese Academy of Agricultural Sciences, National Key Facilities for Crop Genetic Resources and Improvement, Beijing, China; 4 Global Maize Program, International Maize and Wheat Improvement Center (CIMMYT), Carretera, Mexico-Veracruz, El Batan, Texcoco, Mexico; University of Umeå, Sweden

## Abstract

Understanding of genetic diversity and linkage disequilibrium (LD) decay in diverse maize germplasm is fundamentally important for maize improvement. A total of 287 tropical and 160 temperate inbred lines were genotyped with 1943 single nucleotide polymorphism (SNP) markers of high quality and compared for genetic diversity and LD decay using the SNPs and their haplotypes developed from genic and intergenic regions. Intronic SNPs revealed a substantial higher variation than exonic SNPs. The big window size haplotypes (3-SNP slide-window covering 2160 kb on average) revealed much higher genetic diversity than the 10 kb-window and gene-window haplotypes. The polymorphic information content values revealed by the haplotypes (0.436–0.566) were generally much higher than individual SNPs (0.247–0.259). Cluster analysis classified the 447 maize lines into two major groups, corresponding to temperate and tropical types. The level of genetic diversity and subpopulation structure were associated with the germplasm origin and post-domestication selection. Compared to temperate lines, the tropical lines had a much higher level of genetic diversity with no significant subpopulation structure identified. Significant variation in LD decay distance (2–100 kb) was found across the genome, chromosomal regions and germplasm groups. The average of LD decay distance (10–100 kb) in the temperate germplasm was two to ten times larger than that in the tropical germplasm (5–10 kb). In conclusion, tropical maize not only host high genetic diversity that can be exploited for future plant breeding, but also show rapid LD decay that provides more opportunity for selection.

## Introduction

Maize has adapted to different environments with its spread from the center of origin in Mexico to various parts of the world and its evolution into a cultivated and productive plant for food, feed, and industrial products. Depending on the latitude and the environment in which it is grown, maize can be mainly classified into two distinct types, tropical and temperate maize, plus some subtropical lines [1]. Significant differences in artificial and natural selection involved in maize domestication and improvement have contributed to the adaptation of maize to different agro-climatic zones. Detailed knowledge of maize germplasm, particularly temperate and tropical types, is important not only for parental selection but also for genetic analysis and breeding. Molecular markers have been used to construct “phylogenetic” trees and define potential heterotic groups in maize germplasm [2]–[5], establish correlation between genetic distance/combining ability and hybrid performance [6], [7], and assess genetic diversity and understand the population structure at the DNA level in maize landraces, and open-pollinated varieties and inbred lines [8], [9].

The genome-wide genetic diversity and population structure analysis has been largely based on random markers that are developed with limited information on their positions [3], [9]. Genetic variation in different regions of a gene and between genes varies greatly in terms of their extent and associated functions. Understanding of the contribution of genic and intergenic diversity to genetic and functional variation at the genome level needs to use whole genome sequences or marker chips containing a large number of SNPs from different regions of genes. With the advent of next generation sequencing technology, maize inbreds can be resequenced and large allelic regions from many germplasm accessions can be compared [10]. Such studies indicate that the nature of observed allelic differences within genes and their immediate vicinity is different from those within intergenic regions [11]–[13]. Intergenic regions differ dramatically among inbreds and are filled with large insertions and deletions frequently resulting from activities of retrotransposons and DNA transposons [14]. The analysis of intraspecific diversity has to include the understanding of these elements and their impacts on genetic diversity in intergenic regions. Different PCR primers have been designed to target specific regions of previously sequenced genes in maize [15], with the highest level of gene diversity estimated by intron markers (0.46), followed by exon markers (0.42), and promoter markers (0.28). The SNP chips developed recently in maize contain a large proportion of markers from genic regions [16], [17], providing a unique opportunity for understanding the contribution of genic and intergenic variation to genetic diversity and population structure.

With high throughput genotyping and dense SNP maps, the sliding window approach becomes appealing for analyzing haplotypes, with several groups explored this approach from statistical and applied aspects [18]–[20]. In this approach, windows of varying size have been examined, by beginning with the first SNP and moving windows down the framework map without overlapping. This strategy is simple and efficient in comprehensively screening a dense region of markers for association with the trait of interest. A newly developed tool, Graphical Assessment of Sliding *P*-values [21] makes it easier to visualize, summarize and prioritize regions of interest from sliding window haplotype analysis than before, and thus has been used to identify five regions with strong evidence for association with asthma in human.

Genetic diversity has certain relationship with linkage disequilibrium (LD) decay, which in turn affects the diversity and LD-based association mapping. Factors affecting the rate of LD decline with distance include founder effect, mutation, population, selection, mating patterns, genetic drift and migration [22], [23]. As an outcrossing species with large effective population size, maize is expected to have a rapid LD decay. However, the observed LD strongly depends on the population and genomic regions analyzed, with LD decay to half of its starting value within a few hundred bp [24] to a few hundred kb [25], [26]. Analysis of the degree of kinship, amount of ancestral recombination and LD among germplasm collections allows the researcher to choose the most appropriate collection for LD or association mapping.

Selection in breeding programs has significant contribution to various levels of genetic diversity and population structure. On one hand, breeding for heterotic hybrids in temperate maize, mainly in industrial countries, has contributed to the increased genetic difference between heterotic groups, although within-group genetic diversity is relatively low [3], [8], [9]. On the other hand, however, breeding for wide adaptability in tropical maize, mainly in developing countries, has resulted in diverse tropical elite inbreds [4]. LD breakdown and genetic diversity has been studied in elite European maize germplasm [27], and LD broke down significantly slower in elite US maize inbreds than it did in a larger and more diverse sample of maize populations [24]. Among examples, Wang et al used 145 genome-wide SSR markers to assess the genetic diversity, population structure, and LD in 95 Chinese maize inbreds [2]. Yan et al measured LD overall and within chromosomes, subgroups determined by geographic origin, and subgroups of different sample sizes using 1229 SNPs [16]. A comparative analysis using maize inbreds developed from tropic and subtropic regions compare with temperate inbreds is required for a better understanding of germplasm diversity, population structure and LD and their use in genetic improvement.

In our previous study, a total of 447 inbred lines were selected from diverse maize breeding programs to represent tropical and temperate maize diversity [4]. In this study, we applied two Illumina GoldenGate assay chips each containing 1536 SNP markers to genotype the 447 lines. The SNPs in both chips were designed from candidate genes with known or unknown function and intergenic sequences. This kind of high-throughput SNP chips provides opportunities for large scale diversity analysis, high density linkage map construction, LD analysis and genome-wide association studies (GWAS). However, as the most abundant sequence variations found in plant genomes, SNPs are a marker type of choice that best fit our requirements but are limited by the two alleles per locus. Although theoretically each SNP locus has four different alleles, only two of them can be distinguished or designed for genotyping in a specific microarray analysis [4], [5], [16]. This limitation can be overcome by using haplotypes that are constructed from multiple SNP loci [28]. Comparative SNP and haplotype analysis will provide more insights on genetic diversity and relationships among maize lines than any one alone. On the other hand, levels of genetic diversity within and between genes have not been well investigated. With multiple SNPs developed within each gene and gene interval, it has now become possible to compare the genetic diversity contributed by genic and intergenic variation, providing useful information for designing a best set of gene-based markers for molecular breeding.

With SNP markers and their haplotypes developed from different genic and intergenic regions and between genes, this study was designed to: (1) compare the genetic diversity between temperate and tropical maize collections and their relationship with geographic origin and breeding activities; (2) understand the population and subpopulation structure in all tested lines and their relationship with post-domestication selection; (3) understand distribution of LD across the genome and how its breakdown relates to chromosomes and subgroups of germplasm of geographic origins. The information generated in this study will help characterize the genetic diversity and LD in the two major types of maize germplasm that are important to maize genetics and breeding.

## Results

### Summary information of SNPs and haplotypes

From two 1536-SNP chips, 1943 SNPs that were mapped *in silico* onto the maize genome with unique positions and high quality were selected and used for analysis of 447 maize inbreds. These informative SNPs were well distributed across 10 chromosomes, and the coverage ranged from 124 SNPs on chromosome 10 to 316 SNPs on chromosome 1. Based on the sequence annotation analysis performed on the maize B73 filtered gene set, 273 SNPs are from intergenic regions, while the remaining 1670 SNPs are from intragenic regions which involve 1180 genes. Among the 1670 genic SNPs, 512 were designed from intronic sequence, and 1158 from exonic sequence (Table 1). The detailed information of 1943 SNPs is listed in Table S1.

**Table 1:** Summary information for different types of markers and haplotypes (HP) with two or more SNPs.

Marker type	#Loci	#Allele	#Allele per locus	#SNP per locus
All the SNPs	1943	3886	2	1
Intergenic SNPs	273	546	2	1
Intragenic SNPs	1670	3340	2	1
Intronic SNPs	512	1024	2	1
Exonic SNPs	1158	2316	2	1
3-SNP slide-window HP	645	4054	6.2 (2∼8)	3
10 kb-window HP	376	1619	4.3 (2∼23)	2.6 (2∼10)
Gene-window HP	312	1317	4.3 (2∼23)	2.6 (2∼10)

To extract more useful information from the SNP dataset through creating multiple alleles, different types of haplotype were constructed using 1943 SNP markers. To construct 10kb-window haplotypes, 977 SNPs that were more than 10 kb away from any other SNPs were excluded as ‘unlinked’ markers while the remaining 966 SNPs were used to generate 376 10 kb-window haplotypic loci, each consisting of two or more SNPs. The 376 haplotypic loci, with an average of 2.6 SNPs, contained a total of 1619 alleles ranging from 2 to 23 alleles per locus, with an average of 4.3 alleles (Table 1). The number of alleles (haplotypes) at a haplotype locus generally increased with the number of SNPs involved.

When SNPs from the same genes were grouped into one haplotype, 1180 gene-window haplotype loci were constructed using 1670 intragenic SNP markers. Among these 1180 loci, 312 loci, each containing two or more SNPs, were used to compare with other types of haplotypes for genetic diversity. Gene-window haplotypes provided almost the same numbers of alleles and loci as the 10kb-window haplotypes. In addition, 645 3-SNP slide-window haplotype loci were identified each containing 2∼8 alleles averaged 6.2 alleles per locus (Table 1).

### Genetic diversity as revealed by SNPs and haplotypes

With the 447 inbred lines, two alleles were detected at each of the 1943 marker loci. The average PIC was 0.251, ranging from 0.003 to 0.375 with a peak distribution around 0.350 (Table 2). The PIC for 512 intronic SNPs (0.259) was higher than that for 1158 exonic SNPs (0.247). The diversity among 447 lines revealed by the three types of haplotypes varied greatly, and the largest diversity was discovered by 3-SNP slide-window haplotypes, with average PIC of 0.566, followed by the 10 kb-window haplotypes (0.437) and gene-window haplotypes (0.436) (Table 2). The PIC values estimated by the haplotypes (0.436–0.566) were generally much higher than those estimated by individual SNPs (0.247–0.259). The average window size for 3-SNP slide-window was 2160 kb, ranging from 94 bp∼23813 kb, while the average window size for gene-window was 823 bp, ranging from 21 bp∼11.13 kb. The biggest window size that the 3-SNP slide window haplotypes had can be used to explain the highest genetic diversity they identified. Although gene-window haplotypes had much smaller average window size than 10 kb-window haplotypes, they identified the same level of genetic diversity.

**Table 2:** Polymorphic information content (PIC) values estimated by different types of SNP markers and haplotypes (HP) in the entire, temperate and tropical germplasm sets.

	PIC		
Marker type	Entire set	Temperate set	Tropical set
1943 SNPs	0.251	0.228	0.240
273 intergenic SNPs	0.256	0.229	0.251
1670 intragenic SNPs	0.251	0.224	0.238
512 intronic SNPs	0.259	0.239	0.245
1158 exonic SNPs	0.247	0.224	0.235
3-SNP slide-window HP	0.566	0.473	0.548
10 kb-window HP	0.437	0.366	0.425
Gene-window HP	0.436	0.367	0.421

### Comparison of diversity between germplasm sets

The average PIC value among 287 tropical lines was much higher than that among 160 temperate lines, which was consistent across all marker sets, including all SNPs, inter- and intra-genic SNPs, and intronic and exonic SNPs (Table 2). Thus, the tropical germplasm had substantial higher genetic diversity than the temperate.

Table 2 also shows that the PIC values estimated using intergenic and intronic SNPs were higher than those using intragenic and exonic SNPs, which was true for the entire set of 447 lines, tropical and temperate germplasm sets. Compared to the 10 kb-window haplotypes and gene-window haplotypes, 3-SNP slide-window haplotypes provided the highest estimate of PIC across all tested maize lines and each of the two germplasm sets. Gene-window haplotypes and 10 kb-window haplotypes provided similar estimates of PIC.

### Population structure in the tested maize lines

A neighbor-joining tree based on Roger's genetic distance was constructed to gain insight of the genetic diversity in different maize germplasm. The 447 tested lines were grouped into two major groups (Figure S1). In one major group, 163 Chinese lines representing temperate germplasm were grouped along with two CIMMYT lines, and further classified to six subgroups. One subgroup contained 10 maize lines from Southwest China with tropical germplasm background, six landraces and four inbreds developed from landraces. Other five subgroups included Lancaster, Sipingtou (SPT), PA (group A germplasm derived from modern US hybrids), PB (group B germplasm derived from modern US hybrids), and BSSS (Iowa Stiff Stalk Synthetic population), corresponding to five major empirical germplasm origins in China. The clustering result is consistent with heterotic groups established based on the pedigree information and breeders' experience on inbreds' combining ability. The second major group representing tropical germplasm, comprised of 278 inbred lines largely from CIMMYT and four lines from China with tropical background, including A318, 81565, TZI8 and Shuang M9. A318 was developed from S37 that was selected from Suwan population. Line 81565 was developed from Mexican synthetic population, TZI8 from Nigerian inbreds and Shuang M9 from CIMMYT population, Pop28. Unlike the first major group, these 282 lines could not be further divided into subgroups that can be explained based on their environmental adaptation, kernel color, kernel texture, or heterotic performance.

### LD and LD decay across the maize genome and germplasm sets

LD across 1715 SNPs with minor allele frequency (MAF) >0.05 was investigated among the entire (447 lines), temperate and tropical sets. In the 447 inbred lines, LD was significant at the 0.01 level between 34.0% of the pairwise SNPs, in which 9.9% of the pairwise SNPs had the *R* ^2^ more than 0.1 (Table 3). In the temperate set, about 34.1% of pairwise SNPs across 10 chromosomes showed LD, and 59.9% of the significant pairwise SNPs had the *R* ^2^ value more than 0.1. In the tropical set, only 5.6% of pairwise SNPs showed LD, and 10.0% of the significant pairwise SNPs had an *R* ^2^ value more than 0.1.

**Table 3:** Percent pairwise SNP markers in linkage disequilibrium (LD) at *P* = 0.01 level in different (entire, temperate and tropical) sets of maize germplasm.

Chr	#SNPs	Pairwise SNPs in LD (%)			Pairwise SNPs in LD with *R* ^2^>0.1 (%)		
		Entire	Temperate	Tropical	Entire	Temperate	Tropical
1	279	27.7	27.5	3.9	7.9	58.4	6.9
2	206	31.9	32.7	5.6	9.2	58.9	8.8
3	194	34.9	34.7	5.3	8.4	53.7	7.3
4	170	39.9	36.4	5.2	14.7	63.4	12.8
5	223	32.8	37.1	6.0	10.1	62.1	9.3
6	115	32.9	35.2	5.1	9.1	57.1	8.2
7	130	32.3	34.0	7.1	10.0	60.8	11.7
8	173	42.8	37.5	8.0	10.1	56.0	13.2
9	114	38.2	40.1	6.4	11.6	66.4	11.4
10	111	46.4	46.9	6.8	13.9	64.1	17.7
All	1715	34.0	34.1	5.6	9.9	59.9	10.0

The extent of LD across 10 chromosomes had distinct differences. The percentage of pairwise SNPs in significant LD on chromosome 10 in the temperate set (46.9%) was higher than others, and 64.1% of the significant pairwise SNPs exhibited the *R* ^2^ values exceeding 0.1 (Table 3). The percentage of pairwise SNPs in LD on chromosome 1 was the lowest (27.7%) and only 7.9% of the significant SNPs had the *R* ^2^ values more than 0.1 for the entire set. The proportion of pairwise SNPs with significant LD on the remaining chromosomes ranged from 30%–40%. The result indicated that the extent of LD across chromosomes did not correlate with the number of SNP loci involved. Chromosomes 6, 9 and 10 had less SNP loci (<120 loci each), while Chromosome 1, 2 and 5 had more SNP loci (> 200 loci each) (Table 3).

**Figure 1:**
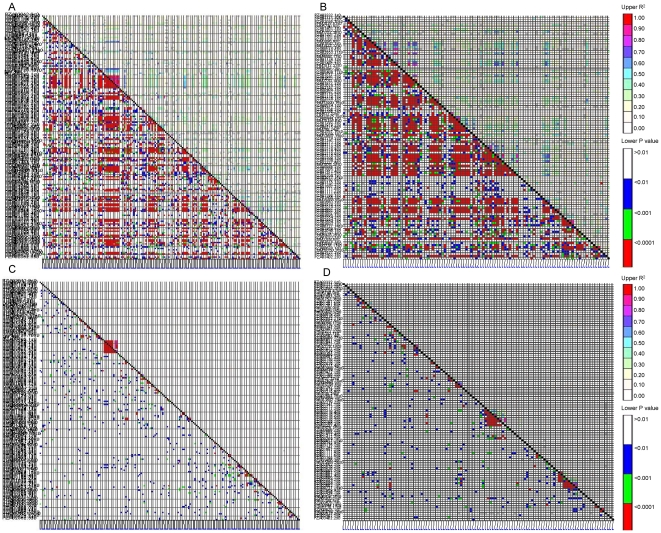
Linkage disequilibrium (LD) patterns on chromosomes 4 and 10 in temperate maize germplasm set (A and B) and tropical maize germplasm set (C and D) genotyped with 170 and 111 SNPs, respectively. The squared correlation coefficients (*R* ^2^) for each pair of markers are presented in the upper triangle and their corresponding tests in the lower triangle: white *p*>0.01, blue 0.01>*p*>0.001, green 0.001>*p*>0.0001 and red *p*<0.0001. The SNPs on each chromosome were aligned up from the left to right long the x-axis and their names and other information can be found from Table S1.

The extent of LD also varied greatly from region to region within chromosomes. Figure 1 showed LD patterns on chromosomes 4 and 10 that had several significant big LD blocks. The significant pairwise SNPs on chromosomes 4 and 10 in temperate set (Figure 1A and 1B, respectively) distributed much more evenly than tropical set (Figure 1C and 1D). On chromosome 4, there was one distinct LD block with P<0.0001 and R^2^≥0.9 in tropical maize (Figure 1C), which spanned around 200 kb and contained nine SNPs. On chromosome 10, two big LD blocks with P<0.0001 were clearly shown in tropical set (Figure 1D). The first LD block spanned around 2 Mb and included 12 SNPs and the second big LD block spanned around 1 Mb including nine SNPs.

With the entire germplasm set (447 lines), LD decay distance for *R* ^2^ greater than 0.1 varied greatly across chromosomes with 1.5–2 kb on chromosome 1, 2–5 kb on chromosomes 5 and 6, and 5–10 kb on the remaining seven chromosomes, with an average of 5–10 kb. The average LD decay is similar with that observed in 632 maize inbreds [16], but a little greater than the previous estimates reported by Remington et al. [29]. Great LD variation among chromosomes implies that LD decay estimation could be biased if the estimation is only based on a single chromosome or a limited number of loci.

**Figure 2:**
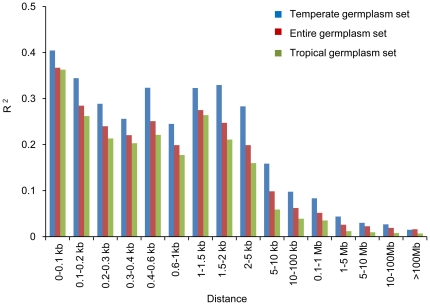
Mean *R* ^2^ over different physical distances for temperate, tropical and entire germplasm sets.

The mean *R* ^2^ values pooled over all 10 chromosomes for the entire, temperate and tropical maize germplasm sets were calculated and shown in Figure 2. In each of three maize germplasm sets, mean *R* ^2^ gradually declined with increased physical distance, similar with previous studies [16], [30]. The LD decay distance for *R* ^2^ to decrease to 0.1 in temperate set (10–100 kb) was much larger than in tropical set (5–10 kb), the former being 10 times larger than the latter. In conclusion, the percentage of pairwise SNPs in significant LD demonstrated a much lower extent of LD in tropical maize than in temperate maize, which was consistent with the result that LD broke down significantly slower in temperate maize than it did in tropical maize.

## Discussion

### Haplotypes and intronic markers as better options for genetic diversity study

SNPs are usually biallelic, each providing less PIC, so that marker density should be increased. Thus, haplotype-based analysis becomes more attractive and important [28]. This study showed a much higher PIC estimated by SNP haplotypes than by single SNPs. However, capturing population structure could be one of the reasons for the higher level of genetic diversity identified by haplotypes, particularly when the haplotypes cover large genomic regions. As a large number of SNP markers are discovered by resequencing [10] or genotyping by sequencing, more genome-wide haplotypes will be developed to characterize the maize genome and used for GWAS and molecular breeding. This has been shown by the first-generation of HapMap [31].

Comparison of large segments of DNA sequence from two *Arabidopsis* genotypes revealed that SNPs occurred at a frequency of 1.4 times greater and indel polymorphisms at a frequency of 3 times greater in introns than in exons. Promoter and intron gene regions have significantly greater length polymorphism within species than exon regions [15]. Intron length variation due to transposable-element indels in introns has also been discovered among alleles of maize genes [32], which can be used to develop allele-specific markers. Our results indicate that intronic markers revealed higher genetic diversity than exonic markers, which was in agreement with the knowledge that exonic sequences are relatively conserved in comparsion with intronic sequences, while intronic sequences evolve rapidly due to the lack of selection pressure, and thus genetic diversity revealed by intronic SNPs increased. As the number of genomic sequence resource increases dramatically for major crop species, a large number of intronic and intergenic markers will become available for genetics and breeding. Sequence-tagged sites can be created for plant-breeding by targeting noncoding gene regions [15].

### High genetic diversity in tropical maize germplasm

We used different types of SNPs and SNP haplotypes to compare genetic diversity between the tropical and temperate maize germplasm sets. Tropical germplasm showed much higher genetic diversity than temperate germplasm as revealed by each type of SNP markers and haplotypes. Haplotype-based diversity analysis not only confirmed previous studies using SSR markers and more recently using SNP markers [8], [16], but also revealed a higher level of genetic differentiation between temperate and tropical maize. It should be indicated that the first SNP chip used in our study is more suitable for diversity analysis of temperate germplasm because the SNPs were mainly developed for maximizing the genetic polymorphisms among temperate inbreds. This bias has been removed largely through selective use of a subset of SNP markers from this chip that showed similar allele frequencies in both temperate and tropical sets [4]. Using two chips containing more non-preferential SNPs should be less biased, as indicated by the result from this study that tropical germplasm showed much higher level of genetic diversity than temperate maize. Large-scale non-preferential SNP genotyping can be achieved through resequencing [10] or genotyping by sequencing [33].

### Implications of rapid LD decay in tropical maize inbreds

In this study, 1715 SNPs with MAF>0.05 were used to determine the structure of LD in the maize genome with a large, diverse collection of 447 lines. A total of 34.0% and 34.1% of the SNP pairs across the genome exhibited significant LD in the entire and temperate germplasm sets, respectively. However, only 5.6% of SNP pairs showed significant LD in the tropical germplasm set. The average decline of LD distance was 10–100 kb in the temperate germplasm set, which was 2∼10 times larger than that in tropical germplasm set (5–10 kb). This significant difference can be explained by the fact that tropical maize has experienced much more intensive recombination and contain more rare alleles than temperate lines [4], [16].

LD observed in a population is the result of interplay of many factors including linkage, population structure, relatedness, selection and genetic drift [22], [23]. LD generated by linkage is considerably useful for GWAS, but LD generated by population structure and genetic drift would result in spurious marker-trait associations. The effect of selection, mutation and relatedness on LD depends on the population under consideration [2]. A rapid breakdown of linkage-related LD will be favorable for association testing of candidate genes that are located nearby the mapped quantitative trait loci (QTL) and have functional relevance to trait variation [34]. The rapid LD decay in maize (5–10 kb), particularly in the tropical maize inbreds tested in this study, provides an opportunity to map QTL with 1000– to 2000-fold greater resolution than current mapping with F_2_ or recombinant inbred populations. Our results provide a rough picture of the structure of LD in maize at the whole genome level and implication for GWAS. The average distance of LD decay revealed in this study (5–10 kb) with a genome size of 2.3 Gb suggests that 230,000 to 460,000 markers will be needed for whole genome scan. Considering that approximately 80% of the maize genome consists of repetitive sequence and also SNPs can be specifically developed from genic regions based on research targets, the actual number of SNPs required for GWAS can be considerably reduced.

## Materials and Methods

### Plant materials

In a previous study, 770 maize inbred lines representing temperate and tropical maize germplasm were chosen from breeding programs and germplasm collections worldwide based on their phenotypes and origins for molecular characterization [4]. Among them, a core collection containing 447 maize inbred lines was used in the present study. This core collection represents phenotypic variation and origins of diverse breeding programs mainly in Mexico, China, Kenya and Zimbabwe. Among 447 core lines, 280 belong to tropical type mainly from CIMMYT maize breeding programs for Latin America and Africa areas, and 167 belong to temperate type except 7 with tropical background which are commonly used in different maize zones and known Chinese heterotic groups. The names of 447 lines together with their environmental adaptation are listed in Table S2. For comparative analysis, the 447 lines were arranged into two sets, tropical line set (287) and temperate line set (160), based on their pedigree information and previous studies. Total DNA was extracted from young leaves of each of the genotypes using CTAB method [35].

### SNP genotyping

Illumina BeadStation 500 G (Illumina, Inc., San Diego, CA, USA) was employed for SNP genotyping at the Cornell University Life Sciences Core Laboratories Center following the protocols [4], [36]. All lines were genotyped using two 1536-SNP chips via Illumina GoldenGate assay [36]. One chip was designed based on random candidate genes (RA chip), which were chosen to study without any prior knowledge or consideration of the function of the proteins (or RNAs) that they encode. The other chip (DT chip) was designed based on 582 candidate genes of known or suspected function that are likely to be involved in the control of drought response [16]. A certain level of bias in SNP identification would happen with these two chips that affect the genetic diversity and population analysis because of the preferential identification of SNPs distinguishing temperate better than tropical maize with the RA chip and the possible non-neutral property of the DT chip. A detailed list of all SNPs from two chips can be downloaded from http://www.panzea.org/.

### Classification of intergenic, exonic and intronic SNPs

To determine the physical positions of the SNP markers used in this study, the original sequences used to develop these SNP markers were used to carry out a BlastN search against the B73 genome sequence (http://www.maizesequence.org) [37]. Only top blast-hits with an e-value threshold ≤ e-^15^ were considered. In 447 inbred lines, the two chips provided 1943 informative SNP markers of high quality with unique physical positions. Detailed information of these informative SNPs can be found in Table S1. Using annotations of the B73 filtered gene set (http://www.maizesequence.org), we categorized SNPs into those located in genic regions (intragenic) and those that fell between genic regions (intergenic) based on their physical positions. Moreover, intragenic SNPs were divided into exonic and intronic SNPs, which were compared with the intergenic SNP for genetic diversity in different maize germplasm sets.

### Construction of SNP haplotypes

The 1943 SNP markers that were mapped *in silico* onto the maize physical map with unique positions were used for construction of haplotypes. To make great use of the information content of SNP data and also compare levels of genetic diversity revealed by different SNP haplotypes, three types of haplotype were constructed using the 1943 SNPs by following [19], [28], [38]: (1) gene-window haplotypes: SNPs from the same gene were scored as a haplotype locus; (2) 3-SNP sliding-window haplotypes: every three consecutive SNPs, on the same chromosome, were used to construct haplotypes with each SNP used only for one haplotype; (3) 10 kb-window haplotypes: the distance between SNPs was added up and SNPs within a total of 10 kb distance were used to construct haplotypes and assigned to the same locus. Haplotype variation within a haplotype locus was recorded as alleles. If an individual has any missing score for any SNP at a haplotype locus, then the entire locus was treated as missing for that individual.

### Genetic diversity analysis

PowerMarker V3.25 was used to calculate the summary statistics including allele number, allele frequency, heterozygosity, and the polymorphism information content (PIC) [39]. PIC was used to refer to the relative value of each marker with respect to the amount of polymorphism exhibited, and estimated by 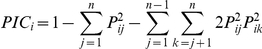
where *P_ij_* and *P_ik_* are the frequencies of the *j*th and *k*th alleles for marker *i*, respectively, and the summation extends over *n* alleles.

To reveal genetic difference using different marker sets, comparative analysis of genetic diversity was performed for inter and intra-genic SNPs, intronic and exonic SNPs, and three types of haplotypes. Diversity statistics for the SNPs and haplotypes were calculated for all the lines and compared with two geographically distinct sets of maize germplasm: tropical and temperate, respectively.

### Genetic structure analysis

The genetic distance between genotypes was computed using the Rogers genetic distances (RD) [40] and cluster analysis was then carried out using the neighbor-joining tree (NJ) method [41]. Groups and subgroups were identified from the resultant phylogenetic tree. All of the above calculations were carried out using PowerMarker software [39].

### LD analysis

LD measurement parameter *R* ^2^ was used to estimate LD between SNPs with MAF>0.05 on each chromosome for the entire germplasm set [2], [16]. In order to see how much the subpopulation would affect the extent of LD, *R* ^2^ was also calculated separately for the tropical and temperate germplasm sets. TASSEL 3.0 software [42], [43] was used to calculate extent of LD (*R* ^2^) between SNP pairs at *P* = 0.01 [2] in the entire set and each germplasm set.

## Supporting Information

Figure S1 Cluster dendrogram constructed for 447 maize inbred lines genotyped with 1943 SNP markers. Two major groups were identified as “Chinese maize lines” and “CIMMYT maize lines”. Six groups were identified within “Chinese maize lines” by different colors as Lancaster, Landrace from Southwest China, SPT, BSSS, PA, and PB
(TIF)

Table S1 Detailed information of SNPs used in the study. The SNP name, chromosome, physical position, SNP type, gene name and ID harboring SNPs, minor allele frequency, polymorphic information content (PIC), and heterozygosity were provided.
(XLSX)

Table S2 List of 447 maize lines used in the study. Information provided in this supplemental table includes brief name, sample name, origin and adaptation.
(XLSX)
