# Supplementary figures and images for: Comparative SNP and Haplotype Analysis Reveals a Higher Genetic Diversity and Rapider LD Decay in Tropical than Temperate Germplasm in Maize
Source: PLoS One. 2011 Sep 15;6(9):e24861. doi: 10.1371/journal.pone.0024861 (PMC3174237; doi:10.1371/journal.pone.0024861)

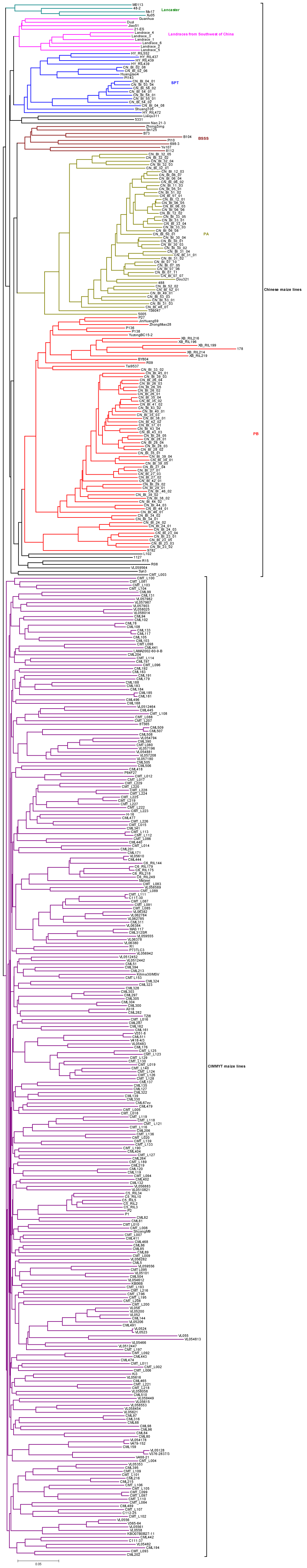

Supplement: Figure S1 — Cluster dendrogram constructed for 447 maize inbred lines genotyped with 1943 SNP markers. Two major groups were identified as “Chinese maize lines” and “CIMMYT maize lines”. Six groups were identified within “Chinese maize lines” by different colors as Lancaster, Landrace from Southwest China, SPT, BSSS, PA, and PB (TIF) [file pone.0024861.s001.tif]
